# Update on Locoregional Therapies for Cholangiocellular Carcinoma

**DOI:** 10.3390/cancers15082368

**Published:** 2023-04-19

**Authors:** Janna Morawitz, Nils-Martin Bruckmann, Kai Jannusch, Julian Kirchner, Gerald Antoch, Sven Loosen, Tom Luedde, Christoph Roderburg, Peter Minko

**Affiliations:** 1 Department of Diagnostic and Interventional Radiology, Medical Faculty, University Dusseldorf, D-40225 Düsseldorf, Germany; 2 Clinic for Gastroenterology, Hepatology and Infectious Diseases, University Hospital Düsseldorf, Medical Faculty of Heinrich Heine, University Düsseldorf, D-40225 Düsseldorf, Germany

**Keywords:** cholangiocellular carcinoma, locoregional therapy, interventional radiology, systemic therapy

## Abstract

**Simple Summary:**

Due to the late onset of symptoms and aggressive growth, cholangiocellular carcinomas (CCA) are associated with poor outcome. In advanced stages, interventional therapies and systemic therapies are particularly used. The combination of locoregional therapeutic approaches with modern system therapies represents a promising approach to improve the outcome for cholangiocellular carcinoma patients.

**Abstract:**

Locoregional therapy options for CCA are used, in particular, for non-resectable tumors and aim to reduce tumor viability or delay tumor growth and ultimately prolong overall survival. In addition to local ablative procedures such as radiofrequency- or microwave-ablation, transarterial procedures such as transarterial embolization (TAE), transarterial chemoembolization (TACE), or selective internal radiotherapy (SIRT) play a major role. In particular, in combination with advances in molecular medicine and immunotherapy, there has been a further development in the therapy of primary malignant liver tumors in recent years. In this review, we analyze data from recent studies and examine the implications for therapy of CCA, particularly with regard to the combination of locoregional therapies with modern systemic therapies.

## 1. Introduction

CCAs can be divided into intrahepatic, perihilar, and extrahepatic cholangiocellular carcinomas. These subtypes differ not only in their anatomic location but also in their incidence, pathogenesis, and treatment. After hepatocellular carcinoma (HCC), CCA is the second most common primary hepatic malignancy, and intrahepatic CCA (iCCA), in particular, has increased over the past years [1,2]. Due to the early invasive growth, but the usually late onset of symptoms, often in the form of painless jaundice, CCA is a major health problem and has a low 5-year survival rate of about 10% [3]. While surgical resection has been the primary curative treatment option in early-stage disease, locoregional therapies and systemic therapy are the leading treatment options for unresectable and locally advanced CCA in more advanced disease stages. Thus, resection is no longer an option for more than 65% of patients at the initial diagnosis [4,5]. Additionally, the fact that approximately 70% of curatively intended resections suffer tumor recurrence means that many patients have to resort to further therapeutic options in the course of the disease [6]. Thus, the complexity of the disease usually requires the interdisciplinary collaboration of multidisciplinary teams. Interventional radiology offers several therapeutic approaches for CCA. In most cases, this involves ablation (radiofrequency ablation, microwave ablation or irreversible electroporation) or transarterial therapies in the form of transarterial (chemo)embolization (TAE and TACE) or selective internal radiotherapy (SIRT). Additionally, hepatic artery infusion (HAI) and, less frequently, chemoperfusion, can be part of a therapy concept. Advances in recent years have revealed promising results regarding locoregional therapy concepts, particularly in combination with modern systemic therapies (e.g. molecular medicine or immunotherapies).

## 2. Percutaneous Tumorablation

In the case of unresectable CCA, local ablative therapy is an alternative option. The most commonly used local ablative procedures include radiofrequency ablation (RFA) and microwave ablation (MWA). In these procedures, a needle is inserted into the tumor percutaneously—guided by sonography or CT—and heated to at least 60 °C, resulting in coagulation necrosis. 

RFA has been an established procedure for the destruction of tumor tissue for several decades and is frequently used for HCC, liver metastases, or tumors in the kidney or lung [7,8,9]. For the ablation of a CCA, RFA was first used in 2002 for an intrahepatic recurrence of a primary extrahepatic CCA [10]. Most of the following studies also focused on non-resectable intrahepatic CCA or on postoperative recurrences [11,12,13,14]. Therefore, RFA can achieve comparable results to re-hepatectomy in recurrence after curatively intended surgery [13]. In primary unresectable intrahepatic CCA, good local tumor control can be achieved if the tumor is no larger than 3–5 cm [15]. However, further studies have shown that RFA is always technically successful for lesions of up to 3.4 cm, but only insufficiently successful for those >4 cm [16]. This shows that tumor size is the most important factor determining the effectiveness and success of RFA. While the ablative margin in other liver lesions such as HCC is described as approximately 2–3 mm [17,18], there have only been a few studies on the ablative margin in CCA, but values of 0.5–1.0 cm have been reported [19,20]. In addition to the possibility of using RFA as an alternative to resection, in individual cases, it is also used as a method for downstaging [21,22]. For CCA, the effectiveness rates for RFA are between 80–100% [20]. Thus, according to a meta-analysis, a pooled local tumor progression of 21% and 1-, 3-, and 5-year overall survival rates of 82%, 47%, and 24%, respectively, were obtained with the use of RFA in the case of unresectable CCA [19]. Based on HCC, single randomized controlled trials that have shown the superiority of combining RFA with TACE or RFA with iodine-125 implantation could be benchmarks for further studies combining RFA with other locoregional therapies [23,24]. Multiple ongoing studies are currently registered to perform RFA at CCA (see Table 1). In particular, combination therapies of RFA with the infusion of cytokine-induced killer cells (NCT02482454) or the combination of RFA with photodynamic therapy (NCT05519319) have been described. The combination of endoluminal RFA with or without endoluminal stenting could also be another therapeutic approach (NCT05563870 and NCT05546372).

Aside from RFA, MWA is another thermal ablation technique that has been increasingly used in recent years. However, studies on sole therapy with MWA are still rare. The technical effectiveness is described with 87.5% and the local tumor progression rate with 25% [25]. With overall survival rates of 93.5%, 39.6%, and 7.9% at 1, 3, and 5 years, respectively, these are comparable to RFA [26]. However, some advantages of MWA over RFA for hepatic lesions have been described. First, MWA seems to be less susceptible to the heat-sink effect, which can negatively affect the ablation outcome, and second, higher intratumoral temperatures have been described for MWA, resulting in more effective, faster, and homogeneous ablation of the tumor [27]. Thus, for HCC, promising results regarding MWA have been described thus far, but no studies exist yet regarding the direct comparison of the efficacy, safety, and outcome of RFA and MWA in CCA.

Other ablation techniques such as cryoablation or irreversible electroporation (IRE) are used less frequently compared with RFA and MWA. A study directly comparing RFA vs. cryoablation in liver malignancies showed no significant differences in local tumor progression, but a higher risk of complications with cryoablation [28]. Studies to investigate the combination of cryoablation in combination with immunotherapy (sintilimab + lenvatinib or camrelizumab) are ongoing (NCT05010668 and NCT04299581). Furthermore, non-thermal IRE can be used in patients unsuitable for thermal ablation, although again, experience seems to be greater for HCC than for other liver malignancies [29]. 

## 3. Transarterial (Chemo-)Embolization (TAE and TACE)

Due to the fact that the liver receives a dual blood supply—75% from the portal vein and 25% from the hepatic arteries - transarterial embolization or transarterial chemoembolization is a progressive approach for local tumor control. Primary liver tumors such as CCA are mainly supplied by the hepatic arteries. Thus, by embolizing the arterial branches supplying the tumor, TAE induces hypoxia in the embolized area, followed by necrosis. In TACE, the technique is combined with the administration of a cytostatic agent to prolong the local residence time and efficacy of the cytostatic agent. 

Patients with locally advanced, unresectable CCA without extrahepatic disease seem most suitable for TAE and TACE. In Europe and the United States, doxorubicin, cisplatin, and mitomycin-C are the most commonly used cytostatic agents for TACE [30,31]. Other options include embolization with drug-eluting beads (DEB-TACE) or with degradable microspheres (DSM-TACE). Both methods are expected to result in a higher drug concentration with lower toxicity. The most commonly used form to date is the conventional lipiodol-based TACE. Together with DEB- and DSM-TACE, it has a varying overall survival between 5.7 and 23 months [32]. A prospective study also showed that the addition of conventional TACE (cTACE) to systemic chemotherapy prolonged overall survival [30], however, another meta-analysis showed no significant differences in terms of the overall survival of cTACE vs. DEB-TACE in CCA. A current prospective randomized phase II study found that the combination of irinotecan-based DEB-TACE (DEBIRI) with cisplatin/gemcitabine-based systemic therapy resulted in significantly prolonged overall survival compared with cisplatin/gemcitabine-based systemic therapy alone (33.7 months vs. 12.6 months, *p* < 0.04) [33]. In addition to prolonging overall survival, further retrospective studies have also demonstrated that TACE in CCA can be a safe and efficacious conversion therapy modality that allows for secondary resectability in initially unresectable CCA [34]. Large randomized controlled trials of the combined use of TACE and immune checkpoint inhibitors have been lacking in CCA. Results from individual HCC trials have demonstrated the superiority of the combination of TACE plus immune checkpoint inhibition (sorafenib) vs. TACE alone [35]. However, various studies investigating the combination of TACE and immunotherapy have also been registered for CCA (see Table 1). For example, the combination of lipiodol-based TACE or d-TACE followed by the oral administration of multi-target drugs (such as lenvatinib or donafenib) or followed by the injection of immunocheckpoint inhibitors (such as sintilimab, tislelizumab, toripalimab, or camrelizumab) is under investigation (NCT05247996, NCT04954781, NCT05448183, and NCT05738057).

## 4. Selective Internal Radiotherapy (SIRT)

SIRT has long been used as a second-line therapy after TACE-failure or in the case of chemotherapy-refractory tumor, but rarely as a primary alternative to TACE. While a German research group already published a study protocol in 2014 for the direct comparison of first-line SIRT vs. first-line DEB-TACE in CCA (NCT01798147) [36], large prospective randomized studies for the direct comparison of these two procedures are still limited thus far. First, promising results of a phase II trial on the use of SIRT in combination with system therapy in locally advanced iCCA were published in 2020. First-line chemotherapy (cisplatin and gemcitabine) was used in combination with ^90^Y-microsphere-based SIRT, and promising results were achieved with a median progression free survival of 14 months, and a progression free survival rate of 55% and 30% at 12 months and 24 months, respectively. In addition, downstaging was possible in 22% of patients, so that R0-resection could be achieved in 20% of all included patients [37]. However, data from a retrospective study were previously published in 2013, showing that overall survival after SIRT appears to be dependent on the tumor phenotype (peripheral vs. infiltrative/15.6 months vs. 6.1 months), number of tumor lesions (solitary vs. multifocal/14.6 months vs. 5.7 months), and tumor burden (<25% vs. >25%/14.4 months vs. 5.3 months) [38]. In retrospective analyses evaluating the time point of SIRT, it has been shown that concomitant SIRT in combination with systemic therapy seems to be superior to the sequential administration of systemic therapy and SIRT (20 months vs. 8.8 months) [39]. Ongoing studies also exist with regard to the combination of SIRT and immunotherapy in CCA (see Table 1). In particular, durvalumab and tremelimumab (NCT04238637 and NCT05655949) have been investigated. In addition, further studies are currently ongoing to investigate the combination of SIRT with established systematic chemotherapy (particularly gemcitabine and cisplatin) (NCT0251269, NCT02807181, and NCT05422690).

**Figure 1:**
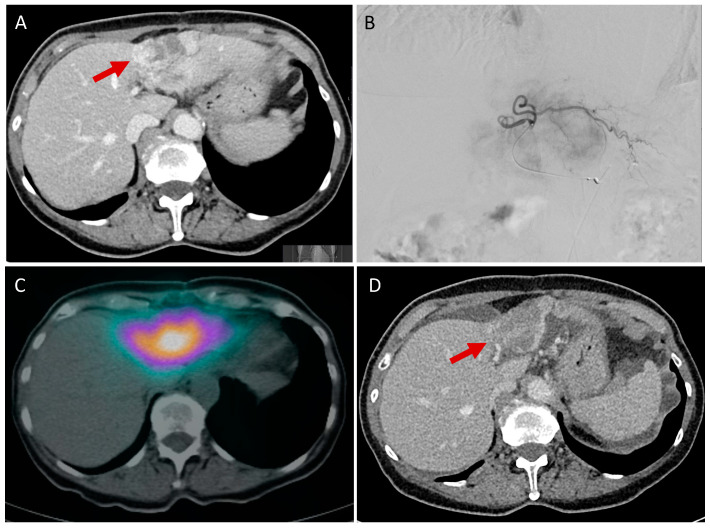
Example of intrahepatic CCA in liver segments II and III with vital tumor portions, as visible by the hypervascularized peripheral tumor parts (**A**, red arrow). After selective internal radiotherapy using 0.44 GBq ^90^Yttrium-loaded particles by catheter positioning in the left hepatic artery (**B**) and acquisition of a SPECT/CT (**C**), postinterventional follow-up 6 weeks after SIRT showed a marked decrease in vital tumor parts (**D**, red arrow).

In addition to the focus on immunotherapy, personalized dose application also plays an important role in the use of SIRT. To maximize tumor response to SIRT while minimizing the dose to non-target tissue, personalized therapeutic activity prescription incorporates patient-specific parameters such as local activity, position, or tissue mass. For example, the recently evolved ZUGSPITZE trial (randomized three arm study, EudraCT 2020-003925-42) is evaluating the radioembolization standard vs. personalized dose + durvalumab/tremelimumab vs. checkpoint first, followed by radioembolization on demand in HCC. Comparable studies for use in CCA do not exist to date. The first studies comparing a standardized ^90^Y dose, calculated on the body-surface-area, and a personalized dose (partition-model), calculated on the basis of tumor to non-tumoral liver-uptake ratio in a previously performed ^99m^Tc SPECT/CT and patient-specific liver, lung, and tumor mass, showed that patients using the partition-model had a significantly prolonged overall survival (5.5 months vs. 14.9 months) [40]. In addition to the partition model, voxel-based dosimetry exists as another type of personalization, which allows the generation of 3D absorbed dose distributions and thus, through dose–volume histograms, the heterogeneity in organs and target tissues can be assessed [41,42,43,44]. International recommendations also provide for the use of the partition-model or the voxel-based model. Moreover, a mean absorbed dose of 40 Gy should not be exceeded for non-tumoral tissue, while the target tissue should receive a dose of 100–120 Gy [45]. For an example of SIRT in CCA, see Figure 1.

## 5. Hepatic Artery Infusion

For hepatic artery infusion (HAI), a durable arterial catheter is surgically inserted into the hepatic arteries to allow for prolonged use for the infusion of local chemotherapy. Via HAI, high concentrations of chemotherapy can be delivered selectively to tumor cells while limiting the toxicity to normal liver cells to a minimum. Cytostatic agents used for HAI should therefore have a high first pass effect, a short plasma-half time, and first-order kinetics with steep dose–response curves with floxuridine being the most favorable [46,47]. Because the first pass effect can limit systemic benefits, HAI is often combined with systemic chemotherapy. Studies of HAI in CCA have also shown that the combination of HAI and systemic therapy is superior to systemic therapy alone (OS 30.8 months vs. 18.4 months) [48]. In large HCC, HAI with oxaliplatin, fluorouracil, and leucovorin showed significant improved overall survival compared to TACE [49], representing a promising approach for HAI therapy in CCA. In HCC, it has already been shown that the combination of HAI with lenvatinib + PD-1 inhibitors led to a significantly better treatment response compared to lenvatinib + PD-1 inhibitors alone [50]. Based on the also proven effect of lenvatinib + PD-1 inhibitors in biliary tract cancers [51], these could be promising results for further studies in CCA.

While response rates of 58%, a progression free survival of 11.8 months, and a 1-year survival rate of 89.5% have been described in a multicenter phase II study using floxuridine in HAI in combination with gemcitabine and oxaliplatin-based systemic therapy [52], studies with comparatively small patient cohorts exist on the use of other cytostatic agents in HAI. Thus, response rates between 16% and 40% have been described for the use of 5-FU, oxaliplatin, cisplatin, or epirubicin [53,54,55,56].

Current ongoing studies on the use of immunotherapy in combination with HAI in CCA are investigating sintilimab and bevacizumab (NCT05400902), tislelizumab and apatinib (NCT05290116), and donafenib (NCT0534881). However, multiple other studies of HAI in CCA are under investigation. In particular, established cytostatics (FOLFOX, cisplatin/gemcitabine or irinotecan, 5-fluorouracil, and leucovorin) are being studied in combination with or in comparison to other therapies such as intratumoral administration of recombinant human adenovirus type 5 (H101) (NCT05124002) or biliary drainage (NCT05024513) (see Table 1).

## 6. Conclusions

Overall, the meta-analyses show that the use of locoregional therapies for CCA are safe and well-tolerated and provide a survival benefit of several months compared to systemic therapy alone [57]. However, studies on locoregional therapies are relatively heterogeneous, often single-center and retrospective, and there is a lack of further prospective randomized studies, particularly when compared to established systemic therapies, in order to make treatment recommendations for CCA more patient-oriented and evidence-based. Therefore, to date, the results are often highly variable with regard to outcome, which is due to the different patient cohorts and procedure-related variables. TACE, TAE, SIRT, and HAI have rarely been compared in randomized trials for CCA, so the decision to use one of these therapies is mostly dependent on local expertise and patient-specific factors. Robust evidence is still lacking to replace the conventional treatments, and large meta-analyses have demonstrated that the current data for locoregional therapies in CCA are currently insufficient to make strong recommendations [58]. However, many ongoing studies exist on the use of locoregional therapies in CCA, especially in combination with immunotherapy, which represents a promising starting point for the further development of CCA therapy.

Already, the published data show a beneficial effect of complementary use of locoregional and systemic therapies in CCA. Both long-established procedures such as ablation and the procedures used in CCA only in recent decades such as TACE and SIRT are currently being further investigated in ongoing and registered trials, particularly with regard to their combination with modern system therapies. Based on the current results of locoregional therapies for HCC and since molecular and immune therapies have clearly changed the treatment of liver malignancies, this represents a promising approach for further studies in CCA. 

## Figures and Tables

**Table 1:** Ongoing trials for locoregional therapy in CCA.

Procedure	Official Title (ClinicalTrials.Gov Identifier)	Study Design	Arm (s)	Recruitment Status
Ablation	Phase III Study of Radiofrequency Ablation Combined with Cytokine-Induced Killer Cells for the Patients with Cholangiocarcinoma (NCT02482454)	Interventional (Clinical Trial) (50 participants)	RFA alone vs. RFA + CIK	Active, not recruiting
Ablation	Clinical Effect and Safety of Photodynamic Therapy Versus Radiofrequency Ablation Versus Photodynamic Therapy Plus Radiofrequency Ablation for Unresectable Extrahepatic Cholangiocarcinoma (NCT05519319)	Interventional (Clinical Trial) (70 participants)	Photodynamic therapy (PDT) vs. radiofrequency ablation (RFA) vs. RFA + PDT	Recruiting
Ablation	A Combined Endoscopy and Radiology-guided Radiofrequency Ablation Therapy Protocol for Inoperable Perihilar Cholangiocarcinoma (NCT05563870)	Interventional (Clinical Trial) (30 participants)	Endoscopic drainage arm vs. COMBO-RFA arm	Recruiting
Ablation	A Phase II Study of Cryoablation Combined with Sintilimab Plus Lenvatinib in Patients with Advanced Intrahepatic Cholangiocarcinoma (CASTLE-01) (NCT05010668)	Interventional (Clinical Trial) (25 participants)	Cryoablation in combination with sintilimab plus lenvatinib	Recruiting
Ablation	A Phase II Study of Cryoablation Combined with Anti-PD-1 Antibody (SHR-1210) in Patients with Advanced Intrahepatic Cholangiocarcinoma (NCT04299581)	Interventional (Clinical Trial) (25 participants)	Cryoablation in combination with camrelizumab	Recruiting
Ablation	Endobiliary Radiofrequency Ablation for Malignant Biliary Obstruction Due to Perihilar Cholangiocarcinoma: A Randomized Controlled Trial (NCT05546372)	Interventional (Clinical Trial) (98 participants)	Endobiliary RFA + stent placement vs. stent placement only	Recruiting
Ablation	Prospective Evaluation of the Ablation Therapy with Bipolar Radio Frequency for Nonresectable Bile Duct Cancer (NCT03679338)	Interventional (Clinical Trial) (20 participants)	Ablation therapy with bipolar radio frequency	Active, not recruiting
TACE	TACE Combined with “Target Immune” Therapy for First-Line Treatment Compared with Intravenous Chemotherapy in the Treatment of Unresectable Intrahepatic Cholangiocarcinoma: A Prospective, Multicenter, Open, Real-World Clinical Study (NCT05247996)	Interventional (Clinical Trial) (98 participants)	Transcatheter arterial chemoembolization combined with “target immune” therapy	Not yet recruiting
TACE	A Phase II Study of Transcatheter Arterial Chemoembolization (TACE) Combined with Tislelizumab in Patients with Advanced Intrahepatic Cholangiocarcinoma (NCT04954781)	Interventional (Clinical Trial) (25 participants)	TACE in combination with tislelizumab	Recruiting
TACE	A Single-Arm, Open-Label, Multicenter Phase II Clinical Study to Evaluate the Safety and Efficacy of Toripalimab Injection Combined with TACE in the Treatment of Extrahepatic Cholangiocarcinoma (NCT05448183)	Interventional (Clinical Trial) (45 participants)	Toripalimab combined with TACE	Recruiting
TACE	Combined Therapy Using D-TACE, Gemcitabine and Cisplatin Chemotherapy, and PD1 Antibody for Patients with Advanced and Unresectable Intrahepatic Cholangiocarcinoma: A Single-Center, Single-Arm Trial (NCT05738057)	Interventional (Clinical Trial) (22 participants)	Combined therapy using D-TACE, gemcitabine and cisplatin, and camrelizumab	Not yet recruiting
TACE	Single-Arm, Multicenter II Phase Clinical Study of DEB-TACE Combined with Surufatinib and Camrelizumab in the Treatment of Inoperable or Metastatic Intrahepatic Cholangiocarcinoma (NCT05236699)	Interventional (Clinical Trial) (18 participants)	DEB-TACE combined with surufatinib and camrelizumab	Not yet recruiting
TACE	Drug-Eluting Beads Transarterial Chemoembolization Combined with Apatinib and PD-1 Antibody for the Treatment of Intrahepatic Cholangiocarcinoma That Has Progressed after Standard First-Line Chemotherapy (NCT04834674)	Interventional (Clinical Trial) (20 participants)	DEB-TACE combined with apatinib and PD-1 antibody	Not yet recruiting
SIRT/TACE	Selective Internal Radiotherapy Is Superior to Transarterial Chemoembolization for the Treatment of Intrahepatic Cholangiocellular Carcinoma (CCC) (NCT01798147)	Interventional (Clinical Trial) (24 participants)	DEB-TACE (doxorubicin) vs. Y90-SIRT	Status unknown
SIRT	A Traditional Feasibility Study of Gemcitabine, Cisplatin, and 90Y TARE for Unresectable Intrahepatic Cholangiocarcinoma (NCT02512692)	Interventional (Clinical Trial) (6 participants)	90Y TARE with gemcitabine and cisplatin	Active, not recruiting
SIRT	Prospective, Multicenter, Randomized, Controlled Study Evaluating SIR-Spheres Y-90 Resin Microspheres Preceding Cisplatin-Gemcitabine (CIS-GEM) Chemotherapy Versus CIS-GEM Chemotherapy Alone as First-line Treatment of Patients with Unresectable Intrahepatic Cholangiocarcinoma (NCT02807181)	Interventional (Clinical Trial) (89 participants)	Chemotherapy (cisplatin-gemcitabine) vs. radiation: SIRT + chemotherapy (cisplatin-gemcitabine)	Active, not recruiting
SIRT	A Phase II Trial of Induction Gemcitabine, Cisplatin and Nab-Paclitaxel Triplet Chemotherapy Followed by Gemcitabine, Cisplatin, and Radioembolization for the Treatment of Locally Advanced Unresectable Intrahepatic Cholangiocarcinoma (NCT05422690)	Interventional (Clinical Trial) (16 participants)	Gemcitabine, cisplatin and nab-paclitaxel chemotherapy with Yittrium-90	Not yet recruiting
SIRT	Phase II Study of Immunotherapy With Durvalumab (MEDI4736) or Durvalumab and Tremelimumab, Both Combined with Y-90 SIRT Therapy in Advanced Stage Intrahepatic Biliary Tract Cancer (BTC) (NCT04238637)	Interventional (Clinical Trial) (50 participants)	Durvalumab vs. durvalumab in combination with tremelimumab	Recruiting
SIRT	A Single-Arm Phase 2 Study of Y-90 SIRT in Combination with Durvalumab (MEDI 4736) and Gemcitabine/Cisplatin in Locally Advanced, Unresectable or Metastatic Intrahepatic Cholangiocarcinoma (NCT05655949)	Interventional (Clinical Trial) (30 participants)	Gemcitabine + cisplatin + durvalumab + Yttrium-90 selective internal radiation therapy	Not yet recruiting
Hepatic Artery Infusion	Recombinant Human Adenovirus Type 5 Combined With Hepatic Artery Infusion Chemotherapy of FOLFOX in Patients With Intrahepatic Mass-forming Cholangiocarcinoma: a Single-site, Single-arm, Prospective Study (NCT05124002)	Interventional (Clinical Trial) (66 participants)	Recombinant human adenovirus type 5 (H101) + HAIC (FOLFOX)	Not yet recruiting
Hepatic Artery Infusion	Hepatic Arterial Infusion Chemotherapy Combined with Sintilimab and Bevacizumab in the Treatment of Unresectable Intrahepatic Cholangiocarcinoma: A Prospective, Single-Center, Phase II Study (NCT05400902)	Interventional (Clinical Trial) (17 participants)	HAIC combined with sintilimab and bevacizumab	Recruiting
Hepatic Artery Infusion	Hepatic Arterial Infusion Chemotherapy Combined with Tislelizumab and Apatinib in the Treatment of Unresectable Intrahepatic Cholangiocarcinoma: A Prospective, Single-Center, Phase II Study (NCT05290116)	Interventional (Clinical Trial) (17 participants)	HAIC combined with tislelizumab and apatinib	Recruiting
Hepatic Artery Infusion	Randomized, Controlled Study to Compare the Efficacy, Safety and Pharmacokinetics of Melphalan/HDS Treatment Given Sequentially Following Cisplatin/Gemcitabine Versus Cisplatin/Gemcitabine in Patients with Intrahepatic Cholangiocarcinoma (NCT03086993)	Interventional (Clinical Trial) (295 participants)	Melphalan/PHP vs. cisplatin and gemcitabine	Active, not recruiting
Hepatic Artery Infusion	Hepatic Arterial Infusion of Gemcitabine-oxaliplatin for Second-line Therapy in Non-metastatic Unresectable Intra-hepatic Cholangiocarcinoma: a Multicentric Single-Arm Phase II Study (NCT03364530)	Interventional (Clinical Trial) (40 participants)	Gemcitabine-oxaliplatin regimen	Status unknown
Hepatic Artery Infusion	Hepatic Arterial Infusion Chemotherapy (HAIC) Combined with Donafenib and Sintilimab in First-Line Treatment of Unresectable Intrahepatic Cholangiocarcinoma (ICC): A Prospective, Open-Label, Phase II Study (NCT05348811)	Interventional (Clinical Trial) (32 participants)	HAIC combined with donafenib and sintilimab	Recruiting
Hepatic Artery Infusion	Hepatic Arterial Infusion Chemotherapy of Oxaliplatin, 5-Fluorouracil, and Leucovorin Versus Systemic Chemotherapy of Gemcitabine and Cisplatin for Unresectable Intrahepatic Cholangiocarcinoma (NCT04961970)	Interventional (Clinical Trial) (188 participants)	Hepatic artery infusion chemotherapy vs. systemic chemotherapy	Recruiting
Hepatic Artery Infusion	Hepatic Arterial Infusion Chemotherapy of Irinotecan, Oxaliplatin, 5-Fluorouracil, and Leucovorin Versus Systemic Chemotherapy of Gemcitabine and Oxaliplatin for Unresectable Intrahepatic Cholangiocarcinoma (NCT03771846)	Interventional (Clinical Trial) (188 participants)	Hepatic artery infusion chemotherapy vs. systemic chemotherapy	Status unknown
Hepatic Artery Infusion	A Phase II Study of Hepatic Arterial Infusion (HAI) with Floxuridine (FUDR) and Dexamethasone (Dex) Combined with Systemic Gemcitabine and Oxaliplatin in Patients with Unresectable Intrahepatic Cholangiocarcinoma (ICC) (NCT01862315)	Interventional (Clinical Trial) (55 participants)	No prior chemo or responded/stable with prior chemo vs. patients who have failed systemic therapy vs. patients who have had prior oxaliplatin and have existing neuropathy	Active, not recruiting
Hepatic Artery Infusion	Prospective Multicenter Trial of Biliary Drainage Plus Hepatic Arterial Infusion Chemotherapy Versus Biliary Drainage Plus Best Supportive Care in Locally Advanced Perihilar Cholangiocarcinomas (NCT05024513)	Interventional (Clinical Trial) (127 participants)	BD-HAIC (biliary drainage and HAIC) vs. BD-BSC (biliary drainage and best supportive care)	Recruiting
Hepatic Artery Infusion	A Phase II Study of Induction Systemic mFOLFIRINOX Followed by Hepatic Arterial Infusion of Floxuridine and Dexamethasone Given Concurrently With Systemic mFOLFIRI as a First-Line Therapy in Patients with Unresectable Liver-Dominant Intrahepatic Cholangiocarcinoma (NCT04251715)	Interventional (Clinical Trial) (30 participants)	mFOLFIRINOX, floxuridine-DEX, mFOLFIRI	Recruiting
Hepatic Artery Infusion	Phase II Study Evaluating the Efficacy of M9241 in Combination with Hepatic Artery Infusion Pump (HAIP) and Systemic Therapy for Subjects with Metastatic Colorectal Cancer or Intrahepatic Cholangiocarcinoma (NCT05286814)	Interventional (Clinical Trial) (48 participants)	M9241 + HAIP FUDR and dexamethasone chemotherapy in combination with FOLFOX or FOLFIRI vs. M9241 + HAIP FUDR and dexamethasone chemotherapy in combination with GemOx	Recruiting
